# Research Advances in the Regulation of Fruit Size: An Integrated Perspective of Genetic, Hormonal, Epigenetic, and Environmental Control

**DOI:** 10.3390/biology14121643

**Published:** 2025-11-22

**Authors:** Haidong Bu, Xiaohuan Sun, Yinghui Hu, Guangjun Gu, Yue Yang, Wenquan Yu

**Affiliations:** Key Laboratory of Cold Region Fruit Breeding and Cultivation, Mudanjiang Branch of Heilongjiang Academy of Agricultural Sciences, Mudanjiang 157000, China; buhaidong11@126.com (H.B.);

**Keywords:** fruit size, quantitative trait locus (QTL), plant hormone, transcription factor, epigenetics, environmental regulation, gene editing

## Abstract

Fruit size is a key economic trait in horticultural crops, determined by an integrated network of genetic, hormonal, epigenetic, and environmental factors. This review synthesizes recent advances in understanding this regulation. Genetically, key quantitative trait loci (QTLs) such as *fw2.2*, *fw3.2*, *FAS*, and *LC* control cell number and organ size. Hormones including auxin, gibberellin, cytokinin, and brassinosteroid regulate cell division and expansion. Transcription factors (e.g., YABBY, WOX families) and epigenetic mechanisms (DNA methylation, histone modifications, endoreduplication) provide additional regulatory layers. Environmental factors (light, temperature, water, nutrition) and cultivation practices (pruning, thinning) ultimately shape the final fruit phenotype. Emerging technologies like multi-omics, CRISPR-Cas9 gene editing, and artificial intelligence offer powerful tools for future research and molecular breeding, which aim to decode the complex regulatory network and enhance crop yield and quality.

## Simple Summary

Fruit size, a vital agronomic trait, is orchestrated by interconnected genetic, physiological, and environmental pathways. Key genes and QTLs (e.g., *fw2.2*, *fw3.2*) modulate cell division and expansion, while phytohormones such as auxin, gibberellin, and cytokinin modulate developmental transitions. Transcription factors, including YABBY and WOX families, along with epigenetic mechanisms like DNA methylation, add further regulatory complexity. External factors-light, temperature, water, and nutrients-interact with cultivation practices to shape the final phenotype. Advances in multi-omics, gene editing, and AI are accelerating the dissection of these networks, offering promising strategies for targeted breeding of high-yield, quality fruit crops.

## 1. Introduction

As the reproductive organ of angiosperms, fruit size is a product of long-term natural selection and artificial domestication, exhibiting remarkable diversity. From a cytological perspective, the final fruit size is primarily determined by cell number, cell volume, and intercellular space, parameters that are precisely regulated during the cell division and cell expansion phases of fruit development [1,2]. The fruit size of cultivated crops often far exceeds that of their wild ancestors; for instance, tomato fruit size may have increased hundreds of times during domestication, due to the selection and modification of key genes and their regulatory networks [3,4].

Fruit development models often exhibit S-shaped or double S-shaped growth curves [5]. The S-shaped growth curve is the most common fruit growth pattern, characterized by a unimodal growth rate. It begins with a slow growth phase dominated by cell division, during which fruit volume increases gradually. This is followed by a rapid expansion phase, where cell division ceases and cell enlargement becomes the primary driver, leading to a peak in growth rate. Finally, during the maturation and growth deceleration phase, the growth rate declines and eventually stops. Examples of fruits following this pattern include apricot [6], pear [7], and apple [8]. In contrast, the double S-shaped growth curve involves an initial period of rapid cell division that establishes the fruit’s basic size, after which cell expansion becomes the main contributor to fruit volume increase [9]. Fruits such as peach, blueberry, strawberry, and grape exhibit this developmental pattern [10,11,12,13]. Beyond the S-shaped or double S-shaped growth curves, there is also a triple S-shaped growth. This complex growth model features three distinct growth peaks separated by two periods of growth stagnation, as observed in kiwifruit development [14]. Studies show that the contribution of cell number versus cell volume to fruit size varies among different fruits. For example, differences in fruit size among sweet cherry [15] and avocado [16] cultivars mainly stem from differences in cell number, whereas the fruit enlargement in apple cultivar ‘Grand Gala’ is primarily attributed to increased cell volume [17]. In many cases, both factors act together, as seen in Japanese persimmon [18].

The regulation of fruit size is a typical complex quantitative trait, determined by both genetic and environmental factors. Genetic factors set the blueprint for the potential range of fruit size, while hormone signaling, transcriptional regulatory networks, and epigenetic modifications fine-tune this blueprint. Simultaneously, environmental factors such as light, temperature, water, and nutrition, along with cultivation practices like pruning and fertilization, ultimately regulate the actual fruit phenotype by influencing the plant’s physiological status and resource allocation [19,20].

In recent years, the widespread application of high-throughput sequencing technologies, gene editing tools (especially CRISPR-Cas9) [21,22,23], and multi-omics integrative analysis methods has enabled scientists to systematically identify key genes and loci controlling fruit size genome-wide and to comprehensively analyze their molecular mechanisms [24]. This article aims to synthesize recent advances in the regulation of fruit size. It presents a comprehensive framework spanning genetics, hormonal control, gene function, transcriptional regulation, epigenetics, and environmental and cultivation management. Additionally, it offers insights into future research directions, with the goal of serving as a valuable resource for researchers and breeders in the field.

## 2. Genetic Basis of Fruit Size Regulation

Genetic factors are the intrinsic decisive force controlling fruit size. Within the same genetic background, even under varying environmental conditions, the final organ size remains largely consistent, indicating that organ size is precisely regulated by the plant’s own internal mechanisms [25]. Utilizing genetic populations to discover quantitative trait loci (QTLs) controlling fruit size variation has become a core strategy for unraveling its genetic basis (Table 1).

### 2.1. Research Progress on Major Fruit Size/Weight QTLs

#### 2.1.1. fw2.2

Tomato, as a model plant for fruit development research, has the most extensively studied genetic regulatory network for fruit size. Over ten major QTLs controlling tomato fruit size have been reported. *FRUIT WEIGHT 2.2* (*fw2.2*) was the first major fruit size QTL cloned in tomato, accounting for approximately 30% of fruit weight variation [26]. It encodes a Cell Number Regulator (CNR), belonging to the CNR/FWL gene family. The *fw2.2* protein localizes to the plasma membrane and acts as a negative regulator of cell division; its expression level is negatively correlated with fruit size. In wild species or small-fruited accessions, *fw2.2* expression is high, inhibiting cell division; whereas in cultivated varieties or large-fruited materials, its expression is reduced or altered in timing due to variations in the promoter region, thereby releasing the inhibition on cell division and increasing fruit cell number [27,28]. Based on sequence variations in the upstream regulatory region of the *fw2.2* gene, CAPS and other molecular markers have been developed. Selecting for the “large fruit” allele can significantly increase fruit weight [26,29]. Homologs of *fw2.2* exhibit conserved negative regulatory functions for organ size in species such as pear [30], avocado [31], sweet cherry [32], rice [33], and maize [34], indicating the widespread and ancient function of this gene family in the plant kingdom.

#### 2.1.2. fw3.2

*fw3.2*/*SlKLUH* is another major QTL for tomato fruit weight, accounting for about 19% of the variation [35]. It encodes a cytochrome P450 enzyme (a member of the CYP78A subfamily), homologous to the *KLUH* gene in Arabidopsis. Copy number variation (CNV) at the *fw3.2* locus is the key determinant of its functional divergence. In wild and small-fruited tomato species, the copy number of the *KLUH* gene is low. In contrast, large-fruited cultivated tomatoes exhibit a significant increase in *KLUH* copy number, as a result of tandem duplications. This increase in copy number directly elevates the mRNA and protein expression levels of the *KLUH* gene. The consequent higher KLUH enzyme activity promotes cell division in the carpel wall (the tissue that develops into the fruit), leading to an increase in fruit volume (primarily in locule size) and ultimately the formation of larger fruits [36]. Research suggests that SlKLUH may promote cell proliferation by influencing lipid metabolism or producing a mobile growth signal [37].

#### 2.1.3. Fas and Lc

The *FASCIATED* (*FAS*) and *LOCULE NUMBER* (*LC*) QTLs primarily increase tomato fruit size indirectly by increasing the number of locules (carpels). The *FAS* locus is associated with the *SlCLV3* gene, where a regulatory mutation due to a chromosomal inversion in its promoter region leads to partial loss of function, resulting in enlarged floral meristems and increased carpel number [38]. A key SNP identified within the *fas* gene was used to develop a functional marker, leading to the efficient breeding of large-fruited tomatoes with high locule number [39]. The *LC* locus is associated with SNPs in the non-coding region downstream of the *WUSCHEL* (*SlWUS*) gene; these SNPs affect *SlWUS* expression, thereby regulating meristem size and locule number [40]. *CLAVATA3* (*CLV3*) and *WUS* form the classic *WUS-CLV* feedback loop, playing a central role in maintaining the balance between stem cell proliferation and differentiation [41]. Mutations in these genes lead to enlarged meristems, increased carpel primordia, and ultimately the formation of multi-loculated, large fruits.

### 2.2. Other Related QTLs

*fw11.3*/*CSR* (*Cell Size Regulator*) affects fruit size by regulating cell volume rather than cell number. *CSR* is highly expressed during the fruit cell expansion phase, and gain-of-function variants can lead to significantly enlarged pericarp cells, resulting in larger fruits [42]. Genes like *OVATE*, *SUN*, and *OFP20* primarily regulate fruit shape but are also closely related to final size. *OVATE* encodes an OVATE family protein, serving as a negative regulator of fruit elongation; its mutation leads to pear-shaped fruits [43]. *SUN* encodes an IQD family protein, and its gain-of-function mutation (caused by retrotransposon-mediated gene duplication) significantly promotes fruit elongation [44]. *SlOFP20* can cooperate with OVATE to enhance the pear-shaped phenotype [45]. In melon, CmOFP6-19b inhibits fruit development by negatively regulating genes related to cell division and expansion (such as *CmCDKB2* and *CmEXPA7*) [46].

QTL mapping and genome-wide association studies (GWAS) have been widely applied to dissect the genetics of fruit size in other horticultural crops. Studies in apple have shown that QTLs for fruit weight, transverse diameter, and longitudinal diameter are distributed across multiple chromosomes [47]. GWAS analysis identified 34 quantitative trait nucleotides (QTNs) associated with fruit size traits [48]. Among them, the homolog of the auxin signaling transcription factor *MdARF106* was predicted as a candidate gene [49]. In sweet cherry, QTLs related to fruit weight and size were anchored to four chromosomes [50]. Research found that genes like *PavCYP78A9*, *PavCYP78A6*, and *PavKLUH* regulate sweet cherry fruit size by promoting cell proliferation and expansion, and are under upstream regulation by MADS-box and AP2/ERF transcription factors [51]. In loquat, QTL mapping based on resequencing identified three QTLs associated with fruit weight, and predicted that *EjEIN4* and *EjTRN1* genes are key candidates regulating intraspecific variation in loquat fruit size [21]. In peach, association analysis combined with gene expression identified the expansin-encoding genes *ppa017982m* and *ppa010443m* as associated with fruit weight [52]. In grape, multiple hybrid populations have mapped several QTLs related to fruit size/weight, with some candidate genes being homologous to tomato *CNR* and *CYP78A* [53]. These findings suggest that, despite the diverse fruit types across species, the genetic basis of size regulation exhibits a degree of conservation, particularly regarding the functions of gene families like *CNR/FWL* and CYP78A/KLUH.

**Table 1:** Major QTL for fruit size.

s.n.	Crop	Gene/QTL	Main Function	References
1	Tomato	*fw2.2*	Negatively regulates cell division; encodes CNR protein; affects fruit size.	[26,27,28]
2	Tomato	*fw3.2/SlKLUH*	Positively regulates fruit size; encodes CYP78A subfamily P450 enzyme; promotes cell proliferation.	[35,36,37]
3	Tomato	*FAS/SlCLV3*	Regulates carpel (locule) number; affects fruit size.	[38]
4	Tomato	*LC/SlWUS*	Regulates locule number; affects fruit size.	[40]
5	Tomato	*fw11.3/CSR*	Regulates cell volume; affects fruit size.	[42]
6	Tomato	*OVATE*	Negatively regulates fruit longitudinal elongation; affects fruit shape and size.	[43]
7	Tomato	*SUN*	Promotes fruit elongation; affects fruit shape and size.	[44]
8	Tomato	*SlOFP20*	Cooperates with OVATE to regulate fruit shape and size.	[45]

## 3. Core Role of Plant Hormones in Regulating Fruit Size

**Figure 1:**
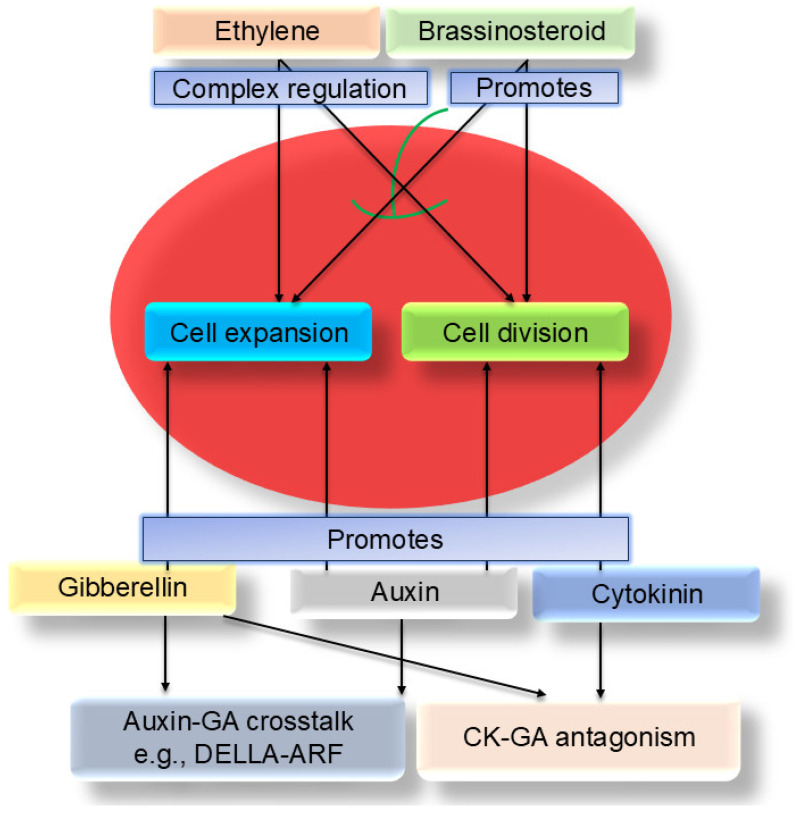
Core phytohormone network regulating fruit size. This diagram summarizes the central roles and complex interactions of major phytohormones in regulating cell division and cell expansion during fruit development. Auxin and Cytokinin (CK) act synergistically to promote cell division primarily during early fruit development. Gibberellin (GA) and Brassinosteroid (BR) are key drivers of cell expansion, with BR promoting both processes. Ethylene plays a complex context-dependent role in early development. These hormonal pathways do not act in isolation but are integrated through extensive crosstalk, forming a sophisticated regulatory network. For instance, Auxin and GA signaling interact via components like DELLA proteins and ARF transcription factors, while CK and GA often exhibit mutual antagonism. This dynamic hormonal balance ultimately determines the final fruit size with precision.

Plant hormones are central signaling molecules regulating fruit development, acting throughout the stages of cell division, expansion, and maturation [19,54]. Auxin, gibberellin (GA), cytokinin (CK), and brassinosteroid (BR) primarily promote cell division and expansion, whereas abscisic acid (ABA) and ethylene are more associated with maturation, senescence, and stress responses but also participate in developmental regulation (Figure 1).

### 3.1. Auxin

Auxin, the first discovered plant hormone, plays a crucial role in fruit set and early development. The synthesis, polar transport (dependent on PIN, AUX/LAX proteins), and signal transduction (dependent on the TIR1/AFB-Aux/IAA-ARF module) of auxin (primarily IAA) constitute a finely tuned regulatory network [20,55]. At low IAA concentrations, Aux/IAA proteins bind to ARFs, repressing downstream gene transcription; at high IAA concentrations, Aux/IAA proteins are ubiquitinated and degraded, releasing ARFs to activate auxin-responsive genes [56]. *SlARF9* negatively regulates cell division in early tomato fruit development; its silencing leads to larger fruits, while overexpression results in smaller fruits [57]. *SlARF7* not only negatively regulates fruit set but also influences fruit development by modulating gibberellin signaling [58]. Silencing *SlIAA9* can induce parthenocarpy and affect fruit weight in tomato [59]. In apple, *MdARF106* is associated with fruit cell division and expansion [49]. MdAux/IAA2 was identified as a negative regulator of fruit and cell size in apple [60].

### 3.2. Gibberellin (GA)

GA primarily influences fruit size by promoting cell elongation. GA biosynthesis involves multiple enzymatic steps, with GA20ox and GA3ox being key synthases, while DELLA proteins are core negative regulators of the GA signaling pathway [61]. When GA is present, it promotes the degradation of DELLA proteins, thereby relieving their repression of growth-promoting genes [62]. DELLA proteins can interact with auxin signaling components like ARFs, enabling crosstalk between GA and auxin signaling [63]. For example, in tomato, silencing *SlDELLA* results in smaller and misshapen fruits and may cause parthenocarpy [64]. Overexpressing *SlGA2ox1* (which degrades active GA) reduces fruit size [65], whereas exogenous application of GA can promote fruit expansion and even induce parthenocarpy [66]. Furthermore, multiple transcription factors participate in the regulation of GA metabolism and signaling. For instance, tomato *SlCDF4* promotes fruit enlargement by regulating GA4 biosynthesis [67]; *SlCRCa* (YABBY family) is involved in feedback regulation of GA biosynthesis, affecting cell division [68]; *SlGAMYB2* positively regulates fruit size by activating *SlGA3ox2* expression and is itself regulated by *SlymiR159* [69].

### 3.3. Cytokinin (CK)

CK primarily regulates cell division. Cytokinin oxidase/dehydrogenase (CKX) is the key enzyme degrading CK. Overexpressing *AtCKX2* in tomato leads to decreased endogenous CK levels, reduced cell division rates, thinner pericarp, and smaller fruits, while also affecting the expression of auxin and GA-related genes, indicating coordinated regulation of fruit development by these three hormones [70]. Exogenous application of CK can stimulate cell division and, in some cases, induce parthenocarpy [71]. In strawberry, trans-zeatin content is high in flower buds and at anthesis, correlating with the spatiotemporal expression patterns of *CKX* genes, regulating the development of the receptacle (the edible part) [72].

### 3.4. Brassinosteroid (BR)

BR promotes both cell division and elongation. BR signaling regulates downstream gene expression through transcription factors like BZR1/BES1. In loquat, *EjBZR1* regulates BR biosynthesis by feedback-inhibiting the expression of *EjCYP90A*, thereby influencing cell expansion [73]. Combined application of exogenous BR and CPPU (a cytokinin analog) can significantly increase grape berry size and cluster weight [74,75]. The functions of BR signaling components can be species-specific. For example, overexpressing tomato *SlBIM1a* (a protein interacting with BES1) results in smaller fruits, whereas overexpressing Arabidopsis *BIM1* increases seed weight [76].

### 3.5. Ethylene and Other Hormones

Although ethylene plays a prominent role in the ripening stage, it also participates in early developmental regulation. Ethylene acts through a series of receptors (e.g., ETR1) and signal transduction components (CTR1, EIN2, EIN3/EIL1, etc.) [77,78]. Tomato *SlTPR1* interacts with ethylene receptors; its overexpression affects IAA and ethylene levels, leading to parthenocarpy and fused fruits [79]. Fruit development is regulated by the synergy or antagonism of multiple hormones. For instance, mutual inhibition exists between CK and GA [80]; ABA-deficient mutants exhibit reduced fruit volume [81]; ethylene influences fruit set by modulating GA levels [82]. These hormones collectively form a complex regulatory network, dynamically guiding normal fruit development [20,83].

## 4. Fine-Tuning of Fruit Size by Transcriptional Regulatory Networks

Transcription factors act as “switches” and “rheostats” in regulating fruit size by binding to cis-regulatory elements in gene promoter regions, thereby activating or repressing the transcription of downstream target genes (Table 2).

### 4.1. YABBY Transcription Factor Family

The YABBY family comprises plant-specific transcription factors crucial for establishing adaxial–abaxial polarity and development of lateral organs. In tomato, the *FAS* locus was initially thought to be a YABBY gene, with its mutation associated with increased locule number [84]. Subsequent fine-mapping revealed that the true causal mutation was a variation in the *SlCLV3* promoter region, while the YABBY gene might be located near or affected by the inversion breakpoint [38,85]. Grape *VvYABBY4* is highly expressed during ovule abortion in seedless varieties; ectopic expression of *VvYABBY4* in tomato leads to dwarf plants and smaller fruits and seeds, suggesting it may affect seed development [86]. SlYABBY2 plays a critical role in regulating the development of roots, leaves, flowers, and fruits in tomato [87]. Specifically, *SlYABBY2a* is expressed predominantly in the fruit septum, and its knockout via CRISPR-Cas9 results in defective septum formation and abnormal fruit morphology. The expression of *SlYABBY2* is regulated by the ripening-related transcription factor SlTAGL1 [22]. Moreover, a mutation at a splice site within the first intron of *SlYABBY2* has been shown to increase ovary number [88].

### 4.2. WOX Transcription Factor Family

The WUSCHEL-RELATED HOMEOBOX (WOX) family is essential for stem cell maintenance and organogenesis. The WUS-CLV pathway: As mentioned earlier, *SlWUS* is the upstream gene of the *LC* QTL; enhanced *SlWUS* expression leads to enlarged meristems and increased locule number, representing a key event in tomato fruit domestication [40,89]. Furthermore, WOX family members are widely expressed in various plant tissues and organs, participating in diverse processes such as embryo development and lateral organ formation [90]. For instance, *WUS* itself plays a central role in maintaining stem cells in the shoot and floral meristems.

### 4.3. Other Important Transcription Factors

Variation in grape *VvNAC26* is associated with fruit size [91], while overexpression of apple *MdNAC1* results in smaller organs [92]. In watermelon, the transcription factor ClNAC100 directly upregulates the expansin gene *ClEXPA1* and gibberellin biosynthesis genes *ClGA3oxs*, thereby promoting plant height and fruit development [93]. In strawberry, the transcription factor FvERF3 directly binds to the promoter of *FvNAC073* to activate its expression, which regulates fruit enlargement and ripening [94]. Tomato *SlPRE2* (bHLH family) influences fruit size by regulating GA metabolism and cell proliferation-related genes; its silencing leads to smaller fruits [95]. Apple *MdANT1* and *MdANT2* (AP2/ERF family) affect early fruit development by regulating cell division [96]. Maize *ZmMYB127* influences kernel size and texture by balancing starch and protein accumulation in the endosperm and interacting with auxin biosynthesis genes [97]. Kumquat *CsMYB77* overexpression delays fruit ripening and results in smaller fruits [98]. RNA-binding proteins: A recent study discovered that the tomato RNA-binding protein SlRBP1 binds to the mRNAs of its target genes *SlFBA7* and *SlGPIMT*, interacts with the eukaryotic translation initiation factor SleIF4A2, and regulates the translation efficiency of its targets, thereby affecting pericarp cell division and expansion. Silencing either *SlRBP1* or its target genes leads to smaller fruits [99]. Tomato STERILE APETALA (SlSAP1 and SlSAP2) F-box proteins form SCF complexes, targeting for degradation the key negative regulators of fruit size SlKIX8 and SlKIX9, thereby positively regulating cell proliferation and expansion. Overexpression of either *SlSAP1* or *SlSAP2* significantly increases fruit size [100]. In soybean, overexpressing *GsMLP328* (Major Latex Protein) enlarges seeds, while knockout has the opposite effect; GsMLP328 interacts with the polygalacturonase-inhibiting protein GmPGIP4 to co-regulate seed size and protein content [101]. In maize, *ZmMYB127* influences kernel texture and size by balancing starch and protein accumulation in the endosperm and interacting with auxin biosynthesis genes, forming a regulatory network [97]. In rice, the transcription factor GS2 activates the expression of *SUG1*, which interacts with multiple transcription factors including OsBZR1, OsMADS56, and OsSPL13, regulating grain size through GA, BR, and growth signaling pathways [102]. A 275 bp deletion in AhARF2-2 disrupts its interaction with AhIAA13 and TOPLESS, thereby reducing the suppression of *AhGRF5* and promoting seed expansion [103]. ENO (EXCESSIVE NUMBER OF FLORAL ORGANS) is a key factor in the floral meristem regulatory network. Acting together with the *LC* and *CLV* pathways, ENO negatively regulates meristem size. Loss of ENO function relieves the suppression of *LC/WUS* expression, resulting in enlarged meristems, increased carpel number, and consequently, larger fruits [104,105]. The MADS-box transcription factor FUL2 (FRUITFULL 2) is essential for normal fruit development, as its double knockout leads to severe developmental defects (Wang et al.). In melon, overexpression of the MADS-box transcription factor CmFYF promotes male flower formation but suppresses fruit size [106]. Furthermore, AS2 (ASYMMETRIC LEAVES 2) and its homolog AS2L (AS2-LIKE) from the LOB family, expressed in the tomato ovary wall, directly control pericarp development by modulating cell layer number and cell area [107].

**Table 2:** Transcription factors of fruit size regulation.

s.n.	Crop	Transcription Factor	Function	References
1	Tomato	SlYABBY2a	Positively regulates fruit septum development and ripening.	[22]
2	Tomato	SlWUS	Regulates meristem size and locule number; a key domestication gene.	[40,89]
3	Tomato	OVATE	Negatively regulates longitudinal fruit elongation; controls pear-shaped fruit.	[43]
4	Tomato	SlCRCa (YABBY)	Involved in feedback regulation of GA biosynthesis, affecting cell division.	[68]
5	Tomato	SlGAMYB2	Positively regulates fruit size by activating *SlGA3ox2* expression.	[69]
6	Tomato	SlPRE2 (bHLH)	Influences fruit size by regulating GA metabolism and cell proliferation-related genes.	[95]
7	Apple	MdARF106	Associated with fruit cell division and expansion (Auxin Response Factor).	[49]
8	Apple	MdNAC1	Overexpression results in smaller organs.	[92]
9	Watermelon	ClNAC100	Directly upregulates *ClEXPA1* and *ClGA3oxs*, promoting plant height and fruit development.	[93]
10	Strawberry	FvERF3	Directly binds to the promoter of *FvNAC073* to activate its expression, regulating fruit enlargement and ripening.	[94]
11	Apple	MdANT1/MdANT2 (AP2/ERF)	Affect early fruit development by regulating cell division.	[96]
12	Grape	VvYABBY4	Ectopic expression leads to smaller fruits and seeds; may affect seed development.	[86]
13	Grape	VvNAC26	Polymorphisms associate with berry size variation.	[91]
14	Kumquat	CsMYB77	Overexpression delays fruit ripening and results in smaller fruits.	[98]
15	Melon	CmFYF	Overexpression promotes male flower formation but suppresses fruit size.	[106]
16	Tomato	AS2 and AS2L	Directly control pericarp development by modulating cell layer number and cell area.	[107]

## 5. Epigenetic Regulation, Endoreduplication and Protein Ubiquitination’s Impact on Fruit Size Determination

**Figure 2:**
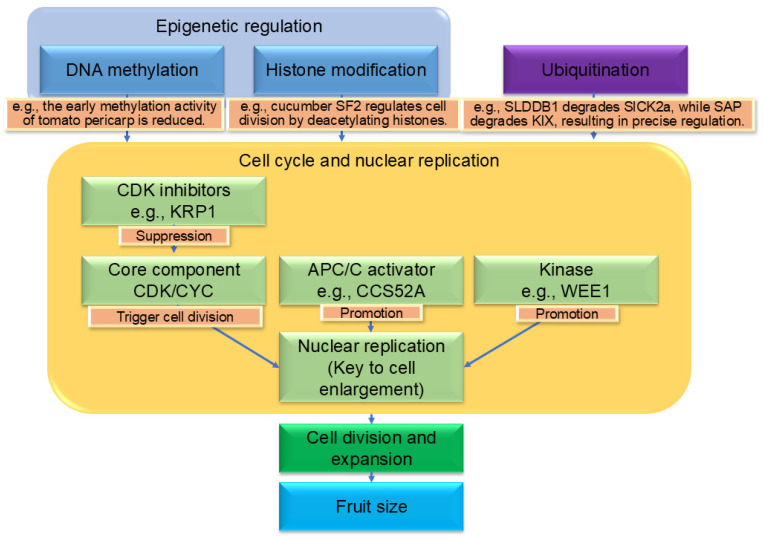
The framework of epigenetic, ubiquitination and cell cycle-mediated regulation of fruit size. This diagram illustrates the core regulatory framework by which epigenetic modifications and ubiquitination processes control fruit size through the cell cycle and endoreduplication. Epigenetic regulation—including DNA methylation and histone modifications—serves as an upstream layer that modulates gene expression without altering the DNA sequence, thereby influencing chromatin accessibility and transcript stability. These epigenetic signals converge on the core execution phase of the cell cycle and the transition to endoreduplication. The cell cycle is driven by CDK/CYC complexes, whose activity is modulated by inhibitors such as KRP. During the cell expansion phase, the cell cycle shifts to endoreduplication (DNA replication without cell division), a process promoted by activators like CCS52A and kinases such as WEE1, ultimately increasing cell volume. Additionally, protein ubiquitination—mediated by complexes such as CUL4-RING E3 ligase (e.g., SlDDB1) and F-box proteins (e.g., SlSAP1/SlSAP2)—targets key cell cycle regulators (e.g., SlCK2α, SlKIX8/9) for degradation, further fine-tuning cell division and expansion. Together, these epigenetic, ubiquitination, and cell cycle mechanisms form an integrated network that precisely controls fruit size.

Epigenetic modifications regulate gene expression without altering the DNA sequence, including DNA methylation, histone modifications, etc., and play significant roles in fruit development (Figure 2). During early tomato fruit development, DNA methylation activity decreases significantly in the pericarp, and this change is tissue-specific [108]. A key DNA demethylase gene, *SlDML2*, has been identified as essential for normal fruit development in tomato. During fruit set and early development, SlDML2 actively removes methylation marks in the promoter regions of multiple genes involved in auxin signaling and cell division. This demethylation activates the expression of these genes, thereby promoting rapid division and expansion of ovary cells. When researchers knocked out *SlDML2* using genetic engineering, DNA methylation levels became abnormally elevated in the plants, leading to the silencing of critical growth genes and ultimately resulting in significantly smaller fruits. This evidence demonstrates that DNA demethylation acts as a key switch triggering and maintaining early fruit development, thereby determining final fruit size [109]. Epigenomic polymorphisms can lead to phenotypic diversity and may be heritable [110]. Through whole-genome DNA methylation sequencing (methylome analysis) of apple varieties with different fruit sizes, significant associations were identified between DNA methylation levels in genomic regions related to fruit size and varietal traits. Hundreds of differentially methylated regions (DMRs) were detected among large- and small-fruited varieties. These DMRs were enriched near genes associated with plant hormone signaling-such as auxin and gibberellin-as well as cell cycle regulation and cell wall modification. This suggests that natural variation in DNA methylation has been fixed by artificial or natural selection during apple domestication and breeding. By regulating the expression of key growth-related genes, these epigenetic modifications contribute to the diversity in fruit size observed among modern cultivated apple varieties [111]. Histone methyltransferase expression increases during the early cell division stage in tomato fruit, indicating active participation of histone methylation in this process [112]. The cucumber short fruit gene *SF2* influences fruit length by affecting the expression of cell division-related genes through histone deacetylation [113]. Endoreduplication is a key feature of the cell expansion phase. Complexes formed by cyclin-dependent kinases (CDKs) and cyclins (CYCs) are the core engine of the cell cycle. Downregulating *CDKA* or overexpressing *CDKB* both lead to smaller tomato fruits and thinner pericarp [114,115]. The kinase WEE1 promotes endoreduplication by phosphorylating and inhibiting CDKA activity; downregulating *WEE1* reduces the level of endoreduplication and results in smaller fruits [116]. *CCS52A*, as an activator of the APC/C E3 ubiquitin ligase, promotes cyclin degradation, driving endoreduplication. Its loss of function leads to smaller fruits and reduced ploidy [117]. *KRP1*, a CDK inhibitor, when overexpressed as *SlKRP1* in tomato fruit, significantly reduces the endoreduplication level but does not alter the final fruit size, suggesting possible compensatory mechanisms [2,118]. Protein ubiquitination is an important post-translational regulatory mechanism. Research found that tomato SlDDB1 (a core component of the CUL4-RING E3 ubiquitin ligase complex) acts as a negative regulator of fruit size by ubiquitinating and degrading SlCK2α (a kinase that positively regulates cell proliferation), while SlCK2α can phosphorylate and stabilize SlCDK2, thus forming a CRL4-CK2α-CDK2 regulatory module that finely controls cell division homeostasis [119]. Additionally, F-box proteins SlSAP1 and SlSAP2 positively regulate leaf and fruit size by targeting the negative regulators SlKIX8 and SlKIX9 for degradation [100].

## 6. Regulation of Fruit Size by Environmental Factors and Cultivation Management

**Figure 3:**
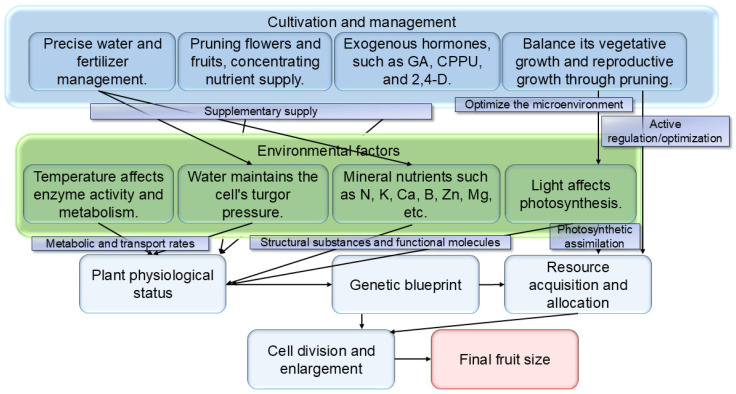
An integrated model of environmental factors and cultivation practices in regulating fruit size. This diagram illustrates an integrated model of how final fruit size is determined by the combined effects of environmental factors and cultivation practices, based on the genetic blueprint. Environmental factors (light, temperature, water, and mineral nutrition) form the physical and chemical basis for fruit growth and development. They directly influence the plant’s physiological status (e.g., photosynthetic rate, metabolic activity, and hydration) and thereby determine resource acquisition. Cultivation practices (pruning, fruit thinning, application of plant growth regulators, and precision irrigation/fertilization) are human interventions designed to optimize the impact of environmental factors (e.g., pruning improves canopy light distribution), directly supplement resources (e.g., fertilization), or manipulate the plant’s resource allocation (e.g., thinning concentrates resources into remaining fruits). These internal and external factors collectively shape the plant’s resource acquisition and allocation system, which ultimately drives the processes of cell division and expansion, realizes the crop’s genetic potential, and determines the final fruit size. This model highlights the scientific rationale behind modern fruit production, which employs integrated management strategies to synergize environmental and cultivation factors for maximizing fruit yield and quality.

The realization of final fruit size depends not only on intrinsic genetic programs but is also profoundly influenced by external environmental conditions and human management practices (Figure 3). Light is the basis of photosynthesis, directly affecting the synthesis and accumulation of organic compounds. Fruits on the outer canopy or receiving ample light are typically larger [120]. Appropriately increasing light intensity favors increased apple fruit weight and soluble solid content [121]. Temperature affects enzyme activity and metabolic rates. Early spring temperatures significantly influence early fruit growth and final size in peach [122]. Climate warming-induced extension of the growing season has also increased the size of plum fruits at harvest [123]. Water is the medium for cell turgor and material transport. Water stress restricts fruit growth, leading to smaller fruits. Studies on pear [124], apple [125], and Satsuma mandarin [126] all indicate a close relationship between fruit yield/size and plant water status. Mineral elements are components of plant structural materials and functional molecules. Appropriate nitrogen application can increase apple fruit size and market value [127]. Foliar application of potassium fertilizer (at 2.0% concentration) significantly increases pear fruit size and soluble solid content [128]. Trace elements such as Magnesium (Mg), Calcium (Ca), Boron (B), and Zinc (Zn) have also been demonstrated to influence fruit size [129,130,131,132,133].

For cultivated crops, human management practices are key to regulating fruit size. Pruning fruit trees (e.g., open-center, spindle shapes) can improve canopy ventilation and light penetration, balance vegetative and reproductive growth, and thereby increase individual fruit weight [134,135,136,137]. Flower and fruit thinning reduces the number of fruits per unit area, allowing the tree to concentrate nutrients into the remaining fruits; this is a classic and effective measure for increasing fruit size, widely used in crops like kiwifruit [138], peach [139], apple [140] and citrus [141]. Scientific application of exogenous hormones according to developmental stage and demand can regulate fruit development. For example, applying 2,4-D during the mango stone hardening stage can significantly increase final fruit size and yield [142].

## 7. Future Research Directions and Prospects

Although significant progress has been achieved in research on fruit size regulation, many scientific questions remain to be further investigated, and the development of new technologies points the way for future research. Future studies need to further strengthen the integrative analysis of genomic, transcriptomic, proteomic, metabolomic, and epigenomic data to systematically dissect the complete regulatory chain from gene to phenotype. For example, multi-omics studies have revealed complex networks among metabolites, proteins, and transcription factors on giant pumpkin [24], jujube [143] and tomato [144], providing new perspectives for understanding the mechanisms of large fruit formation. In the precise application of gene editing technologies, CRISPR-Cas9 and other gene editing tools provide powerful means for functional validation and trait improvement. Future efforts should more widely apply these tools to various horticultural crops beyond tomato, precisely modifying key QTLs or regulatory genes to rapidly breed new varieties with desired fruit sizes. Regarding the deep mechanisms of hormone balance and signaling crosstalk, how do different hormones coordinate precisely in time and space to control the transition from cell division to expansion? The specifics of the interplay between key signaling proteins, such as DELLA and ARF/IAA, remain to be elucidated. The integration of hormone signals with other pathways (e.g., sugar signaling, environmental signals) is also an important direction. In the dissection of QTL regulatory networks, do multiple QTLs exhibit epistatic effects or form regulatory networks? How do their spatiotemporal expression patterns across different fruit developmental stages collectively determine the final size? Utilizing higher-order interaction populations and single-cell sequencing technologies holds promise for revealing these complex relationships. Concerning environmental adaptability regulation mechanisms, investigating how fruit size regulatory networks respond and adapt under different environmental conditions (e.g., high temperature, drought, low light) is crucial for breeding new stress-resistant and high-yield varieties to cope with climate change. In AI and big data-driven predictive breeding, leveraging machine learning and artificial intelligence to integrate multi-source big data including genotype, phenotype, environment, and cultivation management, and establishing precise prediction models for complex traits like fruit size, will greatly enhance breeding efficiency and enable smart breeding.

## 8. Conclusions

Fruit size is a core trait for crop yield and quality formation, and its regulation involves an extremely complex network composed of genetic, hormonal, transcriptional, epigenetic, and environmental factors. This article systematically reviews research progress in this field: at the genetic level, QTLs represented by *fw2.2*, *fw3.2*, *FAS*, and *LC* have been successfully cloned, revealing the central roles of cell number control and meristem regulation. At the hormonal level, auxin, gibberellin, cytokinin, and brassinosteroid, among others, finely regulate the processes of cell division and expansion through complex signaling pathways and crosstalk. At the transcriptional level, multiple transcription factor families such as YABBY, WOX, NAC, and bHLH constitute upstream regulatory hubs. At the epigenetic level, mechanisms like DNA methylation, histone modifications, and endoreduplication provide an additional layer of regulation. Furthermore, environmental factors like light, temperature, water, and nutrition, as well as cultivation practices like flower and fruit thinning, pruning, and exogenous hormone application, also significantly influence the final fruit size. However, the field still faces many challenges, for instance: the conservation and specificity of regulatory mechanisms across different species require further exploration through comparative genomics and functional studies; the hormone balance network and QTL interaction mechanisms are not fully understood; how environmental signals are perceived and integrated into developmental programs remains to be revealed. In the future, with the continuous development and application of cutting-edge technologies such as multi-omics integration, gene editing, and artificial intelligence, we are poised to more comprehensively decipher the regulatory network of fruit size, thereby providing new targets and strategies for high-yield, high-quality, and stress-resistant crop breeding, ultimately promoting the high-quality and sustainable development of the fruit industry.

## Data Availability

No new data were created or analyzed in this study.
